# Anterior Segment Morphology in Primary Angle Closure Glaucoma using Ultrasound Biomicroscopy

**DOI:** 10.5005/jp-journals-10028-1230

**Published:** 2017-10-27

**Authors:** Tarannum Mansoori, Nagalla Balakrishna

**Affiliations:** 1 Consultant, Department of Glaucoma, Anand Eye Institute, Hyderabad Telangana, India; 2 Director Clinical Research, Department of Biostatistics, National Institute of Nutrition Hyderabad, Telangana, India

**Keywords:** Anterior chamber angle, A-scan biometry, Primary angle closure glaucoma, Ultrasound biomicroscopy.

## Abstract

**Aim:**

To evaluate the configuration of the anterior chamber angle quantitatively and study the morphological changes in the eye with ultrasound biomicroscopy (UBM) in primary angle closure glaucoma (PACG) patients after laser peripheral iridotomy (LPI).

**Materials and methods:**

A total of 185 eyes of 185 PACG patients post-LPI and 126 eyes of 126 normal subjects were included in this prospective study. All subjects underwent complete ophthalmic evaluation, A-scan biometry, and UBM. The anterior segment and angle parameters were measured quantitatively and compared in both groups using Student’s t-test.

**Results:**

The PACG patients had shorter axial length, shallower central anterior chamber depth anterior chamber depth (ACD), and anteriorly located lens when compared with normal subjects. Trabecular iris angle (TIA) was significantly narrow (5.73 ± 7.76°) in patients with PACG when compared with normal subjects (23.75 ± 9.38°). The angle opening distance at 500 pm from scleral spur (AOD 500), trabecular-ciliary process distance (TCPD), iris-ciliary process distance (ICPD), and iris-zonule distance (IZD) were significantly shorter in patients with PACG than in normal subjects (p < 0.0001). The iris lens angle (ILA), scleral-iris angle (SIA), and scleral-ciliary process angle (SCPA) were significantly narrower in patients with PACG than in normal subjects (p < 0.0001). The iris-lens contact distance (ILCD) was greater in PACG group than in normal (p = 0.001). Plateau iris was seen in 57/185 (30.8%) of the eyes. Anterior positioned ciliary processes were seen in 130/185 eyes (70.3%) of eyes.

**Conclusion:**

In PACG patients, persistent apposition angle closure is common even after LPI, which could be due to anterior rotation of ciliary body and plateau iris and overcrowding of anterior segment due to shorter axial length and relative anterior lens position.

**How to cite this article:** Mansoori T, Balakrishna N. Anterior Segment Morphology in Primary Angle Closure Glaucoma using Ultrasound Biomicroscopy. J Curr Glaucoma Pract 2017;11(3):86-91.

## INTRODUCTION

Primary angle closure glaucoma (PACG) is one of the leading causes of blindness in Asians.^1-3^ The prevalence of PACG has been reported to be 1.08,^1^ 1.58,^2^ and 4.32%^3^ in population-based studies in Asian Indian eyes.

The PACG eyes have the following ocular biometric findings: A smaller corneal diameter,^4^ smaller radius of anterior^4^ and posterior curvatures,^5^ a smaller central and peripheral ACD^6^ and smaller AC volume,^6^ thicker and anterior position of lens,^4^ shorter axial length,^4^ and greater ratio of lens thickness/axial length factor.^7^

Laser peripheral iridotomy (LPI) is a prophylactic treatment for PACG. It eliminates relative pupillary block and equalizes the pressure in the AC and posterior chamber (PC). However, post-LPI, angle closure is relatively common in east Asian eyes.^8^ In a population-based survey of Chinese persons >50 years of age, 20% of PAC suspects remain gonioscopically closed even with a patent iridotomy.^9^

Gonioscopy is currently the reference standard for clinical assessment of the AC angle. However, it is a subjective test and it cannot provide information of structures posterior to iris, which may influence the angle width.

Ultrasound biomicroscopy (UBM) enables objective and quantitative estimations of angle anatomy.^10^ It provides visualization of retroirideal structures, such as ciliary body, which cannot be seen on clinical examination. The technology of UBM is based on 50-MHz transducer incorporated into a B-mode clinical scanner. It provides lateral and axial physical resolutions of approximately 50 and 25 μm respectively, and has tissue penetration of approximately 5 mm. The scanner produces a 5 × 5 mm field with 256 vertical image lines (or A-scans) at a scan rate of 8 frames/second.^11^

Previous studies using UBM in Asian Indian eyes have revealed that the plateau iris is one of the causes of persistent angle closure after LPI.^12,13^

We evaluated biometric characteristics of eyes with PACG after LPI, which remain appositionally closed on gonioscopy and compared them with normal eyes with gonioscopically open angles, using UBM and A-scan biometry, in Asian Indian eyes. The effect of age and gender on various A-scan and UBM parameters was also evaluated.

## MATERIALS AND METHODS

In this prospective study, 311 subjects (185 PACG patients post-LPI and 126 normal controls) were enrolled. The study protocol was performed in accordance with the tenets of the Declaration of Helsinki.

After informed consent, all subjects underwent comprehensive ophthalmic examination including review of medical history, measurement of best corrected visual acuity, slit lamp biomicroscopy, intraocular pressure (IOP) measurement with Goldmann applanation tonometry, gonioscopy with Sussman lens, fundus examination, automated perimetry with Humphrey visual field (Humphrey visual field analyzer II, Carl Zeiss Meditec, Dublin, California) using the Swedish interactive threshold algorithm standard program and 24-2 test pattern. Gonioscopy was performed in dimly lit room by a single examiner, with 4 mirror Sussman goniolens and patient’s eye in primary position of gaze and 1-mm slit light beam. Care was taken to avoid the light beam to fall on the pupil to prevent light-induced miosis. The UBM was performed with a UBM 840 instrument (UBM B-scan with UBM model, Appasamy associates/Gantec Corporation, USA) using immersion technique, with a 50-MHz transducer probe.

The A-scan ultrasonography was performed after the UBM to measure axial length, lens thickness, and central ACD with A-scan biometry (Echorule 2 A-scan biometer, Biomedix Opto Technik devices, Bangalore, India). Mean value of 10 measurements of sharply rising echo spikes in the A-scans were considered for analysis. Relative lens position (RLP) (defined as relative position of the center of lens determined by adding the ACD to half the lens thickness and then dividing the sum by axial length. The RLP was multiplied by 10).^14^ Cataract was graded clinically using the lens opacity classification system III (LOCS).^15^

When both eyes of the same patient were eligible, one eye was randomly selected for inclusion in the study. Normal subjects had no family history of glaucoma, IOP < 21 mm Hg, open angles on gonioscopy (scleral spur visible 360° with patient looking in the primary gaze), healthy optic nerve, and normal visual fields.

The PACG was defined as presence of appositional angle closure for 270° or more (posterior trabecular mesh-work not visible during static gonioscopy in more than three-quarters of angle circumference, in primary gaze), IOP > 21 mm Hg, and glaucomatous optic neuropathy (defined as loss of neuroretinal rim or rim notch). All PACG patients had undergone LPI with neodymium: Yttrium-aluminum-garnet laser and had patent iridotomy.

Subjects with history of previous intraocular surgery and patients on pilocarpine eye drops, acute ACG, secondary angle closure, and synechial angle closure glaucoma were excluded.

### Examination Technique

An experienced examiner, who was masked to the patient’s clinical data, performed all the UBM scans in a dark room and the patient in supine position. After topical anesthesia, a plastic eyecup was used to gently separate the lids and the cup was filled with normal saline as a coupling agent. Care was taken to prevent eyecup from compressing the globe, which may cause changes in the angle configuration. Fixation and accommodation were held constant by asking the patient fixate with the contralateral eye at a distant target on the ceiling. The UBM probe was held perpendicular to the ocular structures at the limbal region to be examined. Each eye was examined in the axial section, and radial images of the limbus area in the 4 quadrants (superior, inferior, temporal, and nasal) were assessed for each eye. Radial scans were obtained through a typical ciliary process, which showed its relation to the posterior part of iris. Only the images with a clear view of scleral spur, angle, ciliary body, and half full chord of the iris and anterior surface of the lens were included for the analysis.

Images obtained were graded quantitatively in all the 4 quadrants by an examiner with a special caliper in the UBM software. The scleral spur was identified, based on the differential tissue density between the collagen fibers of the scleral spur and the longitudinal muscle of the ciliary body.

For the sake of uniformity, temporal quadrants UBM measurements of all angles were included in the analysis. The UBM parameters measured in the current study have been defined by Pavlin et al.^10^ All the measurements of linear parameters were expressed in millimeters and angular parameters in degrees.

- Angle opening distance 500 (AOD 500) is the distance between the corneal endothelial surface and the anterior surface of the iris measured on a line perpendicular to the trabecular meshwork at 500 ***]im***from the scleral spur.
- Trabecular iris angle (TIA) is measured with the apex of the angle at the iris recess and the arms of the angle passing through a point on the trabecular meshwork at 500 μm from the scleral spur and the point on the iris perpendicularly opposite.
- Trabecular-ciliary process distance (TCPD) is measured on a line extending from the corneal endo-thelium at 500 μm from the scleral spur passing perpendicularly through iris to the ciliary process.
- Iris thickness 1 (ID1) is measured along the same line as the TCPD.
- Iris thickness 2 (ID2) is measured at 2 mm from the iris root.
- Iris thickness 3 (ID3) is measured at the maximum iris thickness near the pupillary edge.
- Iris-ciliary process distance (ICPD) is the distance measured from the posterior surface of the iris to the ciliary process along the same line as TCPD.
- Iris-lens contact distance (ILCD) is measured along the iris pigmented epithelium from the pupillary border to the point where the anterior lens surface leaves the iris.
- Iris-zonule distance (IZD) is measured along the line of TCPD, from the posterior iris surface to the first visible zonule fiber at the point just clearing the ciliary process.
- Iris lens angle (ILA) is the angle between the iris and the lens near the pupillary edge.
- Scleral-iris angle (SIA) is measured between the tangent to the scleral surface and the long axis of the iris.
- Scleral-ciliary process angle (SCPA) is measured between the tangent to the scleral surface and the long axis of the ciliary process.

Based on UBM images, plateau iris in a quadrant was defined by using standardized qualitative criteria as described before,^13^ i.e., anteriorly directed ciliary process, absent ciliary sulcus, steep iris root from its point of insertion followed by a downward angulation, and irido-angle contact (above the level of the scleral spur) in the same quadrant. At least two quadrants had to fulfill these UBM criteria for an eye to be classified as having plateau iris.

Statistical analysis was performed with commercial software, Statistical Package for the Social Sciences, version 15 (SPSS Inc, Chicago, IL). Comparison of A-scan biometry and UBM parameters between controls and PACG patients were performed with the student’s t-test. Correlation between age and various A-scan biometry and UBM parameters was analyzed using Pearson correlation coefficient. A probability value of < 0.05 was considered significant.

**Table 1:** **Table 1:** Baseline demographics of study participants

*Variable*		*PACG (185)*		*Controls (126)*		*p-value*	
Age (years)		59.77 ± 9.62		57.79 ± 9.84		0.08	
Male (%)		106 (49.8%)		65 (51.6%)			
Female (%)		79 (80.6%)		61 (48.4%)			
Sphere (Diopter)		0.89 ± 2.08		0.06 ± 0.82		0.06	
Cylinder (Diopter)		–0.16 ± 1.02		–0.16 ± 0.16		0.98	

**Table 2:** **Table 2:** Mean ± standard deviation (95% confidence interval) of biometric values of PACG patients and normal control groups obtained using A-scan ultrasound biometry

*Variables*		*PACG*		*Controls*		*p-value*	
Axial length (mm)		22.45 ± 0.98 (22.15-22.75)		23.59 ± 1.03 (23.19-23.99)		<0.0001	
Central anterior chamber depth (mm)		2.26 ± 0.4 (2.16-2.37)		2.83 ± 0.4 (2.72-2.95 )		<0.0001	
Lens thickness (mm)		4.67 ± 0.61 (4.48-4.85)		4.54 ± 0.78 (4.24-4.84 )		0.45	
RLP		2.12 ± 0.01 (2.04-2.19)		2.25 ± 0.02 (2.17-2.34)		0.012	

## RESULTS

A-scan biometry and UBM scans of a total of 185 eyes of 185 PACG patients and 126 eyes of 126 normal subjects were analyzed. The mean age ± standard deviation (SD) of PACG patients was 59.77 ± 9.62 years (42-83 years) and 57.79 ± 9.84 years (36-75 years) in control group. Demographic characteristics of study population are summarized in Table 1.

An early cataract (LOCS III < grade II) was present in 30% of eyes in PACG group and 20% of eyes in control group.

In patients with PACG, the axial length was significantly shorter (22.45 ± 0.98 *vs* 23.59 ± 1.03 mm) and the central AC depth was shallower (2.26 ± 0.4 *vs* 2.83 ± 0.4 mm) than in normal individuals. There was no difference in lens thickness between the two groups; however, the lens was more anteriorly located (RLP) in PACG eyes (Table 2).

The results of UBM parameters are shown in Table 3. The TIA was significantly narrower in patients with PACG when compared with normal subjects. The AOD 500, TCPD, ICPD, and IZD were significantly shorter in patients with PACG than in normal subjects. The ILA, SIA, and SCPA were significantly narrower in patients with PACG than in normal subjects (Table 3).

The ILCD was significantly greater in PACG group than in normal controls. There was no significant difference in iris thickness (ID1, ID2, ID3) between the two groups.

In PACG group, older patients had shallower central ACD (Pearson correlation coefficient (r) =-0.324, p = 0.012), shorter TCPD (r =-0.148, p = 0.04), shorter ICPD (r =-0.216, p = 0.003), and narrower TIA (r =-0.225, p = 0.002). In control group, older patients had shorter TCPD (r =-0.202, p = 0.024) and shorter ICPD (r =-0.266, p = 0.003).

In PACG group, after adjusting for age, females had shorter axial length than men (21.86 ± 0.82 *vs* 22.94 ± 0.83 mm, p < 0.001), shorter TCPD (0.57 ± 0.16 *vs* 0.64 ± 0.18 mm, p = 0.005), hyperopic refraction (+2.05 ± 2.01 *vs* -0.02 ± 1.62 D, p = 0.01), and thinner basal iris (0.39 ± 0.07 *vs* 0.45 ± 0.09 mm, p < 0.0001). All other variables were comparable between the groups. In control group there was no statistically significant difference between men and women.

**Table 3:** **Table 3:** Mean ± standard deviation (95% confidence interval) of ultrasound biomicroscopic parameters of PACG patients and normal control group

*UBM parameters*		*PACG*		*Controls*		*p-value*	
AOD 500*		0.07 ± 0.08 (0.06-0.08)		0.22 ± 0.09 (0.21-0.24)		<0.0001	
TCPD*		0.61 ± 0.17 (0.58-0.63)		0.84 ± 0.18 (0.81-0.88)		<0.0001	
Iris thickness (ID)*							
ID1		0.42 ± 0.09 (0.41-0.49)		0.43 ± 0.08 (0.42-0.45)		0.48	
ID2		0.44 ± 0.08 (0.43-0.45)		0.45 ± 0.08 (0.44-0.47 )		0.06	
ID3		0.58 ± 0.11 (0.56-0.59)		0.6 ± 0.11 (0.58-0.62)		0.9	
ILCD*		0.78 ± 0.31 (0.74-0.83)		0.67 ± 0.23 (0.63-0.71 )		0.001	
IZD*		0.51 ± 0.14 (0.49-0.53)		0.58 ± 0.13 (0.56-0.61)		<0.0001	
ICPD*		0.09 ± 0.12 (0.07-0.1)		0.2 ± 0.14 (0.17-0.22 )		<0.0001	
TIA**		5.73 ± 7.76 (4.60-6.85)		23.75 ± 9.38 (22.09-25.41)		<0.0001	
ILA**		15.06 ± 5.72 (14.22-15.9)		18.2 ± 5.29 (17.26-19.14)		<0.0001	
SIA**		29.77 ± 4.89 (29.05-30.5)		33.67 ± 5.12 (32.77-34.58)		<0.0001	
SCPA**		50.6 ± 8.53 (49.34-51.87)		55.74 ± 10.19 (53.94-57.55)		<0.0001	

*All values are in millimeters; **Values are in degrees

Using predefined criteria, UBM analysis found plateau iris in 57/185 (30.8%) of PACG eyes. Anteriorly directed ciliary processes were detected in 130/185 eyes (70.3%) of PACG patients. However, in the normal eyes also, anterior directed ciliary process was observed in 10/185 eyes (5.4%) with open angles on gonioscopy and UBM.

## DISCUSSION

In PACG patients, post-LPI, residual angle closure due to multiple mechanisms is common in Asian eyes.^12,13^ Compared with normal eyes, the PACG eyes post-LPI examined in our study showed a shorter axial length and shallower central ACD and anteriorly located lens. Shallow AC in this population can also be explained by anterior position of ciliary process, which results in anterior lens position and shorter TCPD, which also represents the relative position of ciliary processes. A small ACD implies smaller AC volume which may facilitate angle-crowding mechanism. These eyes with persistent appositional closure have significant biometric differences (A-scan and UBM) from normal healthy eyes and are at risk for progressive damage to the trabecular meshwork and creeping angle closure.

Garudadri et al^12^ in a cohort of 55 Asian Indian patients with PACG after LPI, using UBM in dim ambient illumination, reported AOD 0.112 ± 0.08 mm in PACG patients and TCPD value of 0.604 ± 0.193 mm in PACG patients. Narrow angle (defined as AOD <130 u) with anteriorly placed ciliary process and a narrow ciliary sulcus were seen in 66.6% of PACG eyes after LPI. They found that the ID1 was thinner in PACG patients post-LPI when compared with the normal.

Using UBM under semidark conditions, Kaushik et al^16^ in 106 Indian eyes reported mean AOD 500 values of 0.1 ± 0.08 and 0.29 ± 0.06 mm in eyes with narrow and open angles respectively. They reported mean TCPD of 0.65 ± 0.12 mm in narrow angles and 0.88 ± 0.09 mm in open angles.

Sihota et al^17^ compared UBM parameters in 30 chronic PACG eyes and 30 normal subjects. The TIA was narrower in PACG eyes when compared with normal and AOD 500, TCPD, ICPD were significantly smaller in PACG eyes when compared with normal controls. In this study, eyes with chronic PACG had a decreased iris thickness as compared with POAG and controls. This speculated that the decrease in iris thickness may be a consequence of ischemic iris atrophy. The ILCD was not significantly different among the groups, with chronic PACG eyes having the maximum ILCD (Table 4).

In a prospective UBM study by our group on 262 PACG patients and 144 normal controls showed plateau iris in 83/262 (31.68%, 95% confidence interval: 26.7-37.9%) of PACG eyes post-LPI.^13^

Previous studies on Asian Indian eyes found thinner iris in their study group of PACG when compared with normal controls.^12,17^ In Eskimos, thicker iris has been reported, which contributes to narrow angle and an increased risk of angle closure.^18^ In our study, there was no difference in the iris thickness in PACG patients and normal controls.

The biometric features, such as TCPD, ICPD, and AOD were similar in other studies on Asian Indian eyes.^12,17^ However, direct comparison cannot be made between these studies^12,16,17^ on Asian Indian eyes, as definition of narrow angle is different in a study; other 2 studies^16,17^ did not measure SIA and SCPA, and the direction of ciliary processes position was not mentioned (Table 4). Additionally, only one of the studies^12^ was on patients with PACG post-LPI, which is similar to our study population.

Marchini et al^14^ in 54 White patients with PACG performed UBM under light conditions and reported mean AOD 500 in PACG patients and normal controls as 0.21 ± 0.1 and 0.36 ± 0.11 mm respectively. They reported AC angle as 19.87 ± 9.83° in PACG and 31.29 ± 9.18° in normal subjects, mean TCPD as 0.71 ± 0.14 and 1.08 ± 0.22 mm in PACG and normal controls respectively. The PACG patients had shorter axial length, shallower AC, and thicker and anteriorly located lens.

**Table 4:** **Table 4:** Summary of UBM parameters in studies on Indian eyes in PACG patients

*UBM parameters*		*Sihota et a?^7^ (n = 30)*		*Kaushik et* al^16^ *(n = 106)*		*Garudadri et* al^12^ *(n = 55)*		*Our study (n = 185)*	
AOD 500*		0.099 ± 0.054		0.102 ± 0.084		0.112 ± 0.083		0.07 ± 0.08	
TCPD*		0.931 ± 0.129		0.653 ± 0.124		0.604 ± 0.193		0.61 ± 0.17	
Iris thickness (ID)*									
ID1		0.395 ± 0.156		–		0.347 ± 0.092		0.42 ± 0.09	
ID2		0.481 ± 0.078		–		–		0.44 ± 0.08	
ID3		0.540 ± 0.091		–		–		0.58 ± 0.11	
ILCD*		1.45 ± 3.31		–		–		0.78 ± 0.31	
IZD*		–		–		–		0.51 ± 0.14	
ICPD*		0.402 ± 0.098		–		–		0.09 ± 0.12	
TIA**		11.24 ± 7.27		–		–		5.73 ± 7.76	
ILA**		–		–		–		15.06 ± 5.72	
SIA**		–		–		24.90 ± 5.00		29.77 ± 4.89	
SCPA**		–		–		35.42 ± 7.245		50.6 ± 8.53	

Amillimeters; **All values are in degrees; - Blank signifies that the values are not measured in the study

In our patients with PACG, the SCPA was significantly narrower than in normal eyes. The TCPD, AOD 500, ICPD, and IZD were shorter in PACG eyes than in normal eyes, indicating a more anterior rotation of ciliary processes, which was found in 70.3% of the eyes. The TCPD is influenced by ciliary processes position, which is determined by SCPA. The anterior position of ciliary processes could be a predisposing factor for the development of creeping angle closure.

In our study, SIA was narrower in PACG group than in normal, a finding which is consistent with that of obliquity of the iris plane. The PACG patients had greater ILCD and narrower ILA when compared with normal, a feature that could be due to more anterior position of the lens and also, the iris falls back after the LPI as pupillary block is relieved. A study by Caronia et al^19^ in 13 White and Hispanic eyes reported that the entire iris falls back with LPI for appositional closure, which results in increase in ILCD.

In a population-based study, older Chinese subjects had a shallower central ACD and shorter axial length as measured by A-scan ultrasonography.^20^ In Liwan Eye Study, based on gonioscopy, older subjects had narrower iridotrabecular angle (≤20°).^21^ Using slit lamp-based optical coherence tomography, the Beijing Eye Study reported that older Chinese people had a narrower AC angle and shallower ACD.^22^ The current study showed that the older subjects probably had more anteriorly directed ciliary body in both PACG and normal groups as indicated by shorter TCPD.

The difference between men and women in the configuration of the anterior ocular segment has been evaluated in several population-based studies. Most studies found that women have shallower central ACD,^20^ shallower peripheral AC angle,^21^ and a thicker crystalline lens.^20^

In this study, we found significant differences in UBM parameters in female patients with PACG post-LPI as having shorter TCPD, thinner basal iris, shorter axial length, and hyperopic refraction when compared with males, after adjusting for age, whereas normal subjects did not show significant intergender differences.

Limitation of the study is that the gonioscopy and quantitative measurements of UBM images were performed by the same examiner, which can cause bias in the results. The quantitative UBM analysis was based only on single cross-sectional image of the temporal quadrant angle and hence, variations in the quadrant may be missed. Strength of the study was that this was the UBM study on eyes with appositional closure post-LPI with shallow AC and no peripheral anterior synechiae.

In summary, our study showed that the appositional angle closure is common after LPI in PACG eyes in this study population, which could be due to anterior rotation of ciliary processes and plateau iris. These eyes are at the risk of developing further angle closure and need close monitoring.
